# Ejaculate collection efficiency and post-thaw semen quality in wild-caught Griffon vultures from the Sardinian population

**DOI:** 10.1186/1477-7827-7-18

**Published:** 2009-02-19

**Authors:** Manuela Madeddu, Fiammetta Berlinguer, Massimo Ledda, Giovanni G Leoni, Valentina Satta, Sara Succu, Andrea Rotta, Valeria Pasciu, Angelo Zinellu, Marco Muzzeddu, Ciriaco Carru, Salvatore Naitana

**Affiliations:** 1 Department of Animal Biology, University of Sassari, Via Vienna 2, 07100 Sassari, Italy; 2 Department of Physiological, Biochemical and Cellular Sciences, University of Sassari, Via Vienna 2, 07100 Sassari, Italy; 3 Presidenza, Biblioteca Veterinaria, Faculty of Veterinary Medicine, University of Sassari, Via Vienna, 2 07100 Sassari, Italy; 4 Department of Biomedical Science, University of Sassari, Viale S. Pietro 43/B, 07100 Sassari, Italy; 5 Sardinian Board of Forestry, viale Luigi Merello, 86 – 09123 Cagliari, Italy

## Abstract

This study aimed to test the feasibility of a programme of semen collection and cryopreservation in Griffon vultures. Four wild-caught individuals kept in captivity because of unrecoverable traumas were used. Semen collection attempts were made twice a week during three consecutive reproductive seasons (December – March) using the abdominal massage method. Ejaculation was successfully induced between late January and late February. Semen collection efficiency was rather low (27.9%) and it did not vary among individuals (p > 0.05). No differences were found in ejaculate volumes (12.5 +/- 9.1 μl), spermatozoa concentration (28.4 +/- 30.9 million cells/ml) and viability (61.3 +/- 13.9%) among the 4 vultures. ATP values differed among the four vultures (p < 0.001); B showed higher nucleotide concentration than both C and D, while it did not differ form A, whose values were higher compared with D. After freezing and thawing, semen in vitro viability, DNA integrity and ATP intracellular concentration were determined. Spermatozoa viability after thawing did not differ among the four individuals (52.6 +/- 5.8 in A, 53.4 +/- 4.6 in B, 50.4 +/- 3.2 in C, 42.5 +/- 2.7 in D), but it decreased significantly compared to fresh semen (p < 0.05). During 4 hrs in vitro culture, spermatozoa collected from B maintained over time a higher viability in vitro when compared to A, C and D. As evaluated by the comet assay method, DNA fragmentation after freezing and thawing did not differ in the 4 vultures. ATP concentration in frozen/thawed semen was significantly lower than in fresh semen (p < 0.0001). This study indicates that semen cryopreservation can be considered as a useful tool in the conservation of Griffon vulture genetic resources, but further studies are needed to optimize this technique.

## Background

Establishing a genome resource bank, along with assisted breeding techniques, has the potential to preserve genetic diversity of many endangered species managed ex situ [1]. Frozen semen and embryos can be used to minimize inbreeding and genetic drift in small populations. Moreover, the subsequent risk of expression of unfavourable or even lethal genetic traits, as well as the loss of advantageous characteristics through unnatural selection, can be lowered [2]. We have reported the potential application of semen cryopreservation in a program of gamete and embryo banking, designed to restore genetic diversity in small and isolated populations of wild ruminants, such as the European mouflon (Ovis gmelini musimon) and endangered gazelle (Gazella dama mhorr), through both in vivo and in vitro reproductive techniques [3-5].

In the case of birds, it is not possible to cryopreserve embryos or oocytes, mainly because of their large size, the high lipid content, and the polar organization [6]. Therefore, the most feasible method for ex situ management of genetic resources in birds is semen cryopreservation. The feasibility of the construction and utilization of an avian semen cryobank for the ex situ management of endangered pheasants and of rare domestic lines and breeds of the species *Gallus gallus *has recently been reported [7,8]. Artificial insemination with frozen and thawed semen has also been used to produce chicks in non-domestic bird species, such as the American kestrel [9], the Sandhill crane [10], the Canadian goose [11], and the Budgerigar [12]. It is noteworthy that researchers have promptly responded to the well known catastrophic collapse in the population of the Indian white-backed vulture (*Gyps bengalensis*) by studying its basic spermatology and developing a protocol for semen cryopreservation in order to conserve the specie through captive breeding and assisted reproduction [13].

Endangered bird conservation efforts can thus rely on the application of assisted reproduction techniques and the integration of long-term intensive demographic and genetic monitoring with the parallel creation of a semen cryobank of the local threatened population would guarantee a "life insurance" for the specie.

In Sardinia, Italy, out of three large vulture species present until the mid-twentieth century (*Aegypius monachus*, *Gypaetus barbatus *and *Gyps fulvus*), only the Griffon vulture (*Gyps fulvus*) has survived until today. Distributed over the whole island up to the late 1940s with an estimated population of 800 – 1200 individuals, the population of Griffon vultures in Sardinia dropped very rapidly after the Second World War, mainly in relation to the use of poisoned baits, outlawed in 1977. In 1975 the estimated population counted only 100 – 140 Griffon vultures [14]. In order to save the last Griffon vulture native population in Italy from extinction, several conservation projects focused on food supplementing and creation of new protected areas have been carried out during the last 30 years. Despite these efforts, different poisoning actions in 1997/98 and the further loss of 30–35 individuals in 2006/07 reduced the population to the present 60–65 individuals and 21–22 breeding pairs, concentrated by more than 90% in the north-west of Sardinia. This situation begs for the development of additional conservation strategies.

The aim of our study was to test the feasibility of a programme of semen collection and cryopreservation in Griffon vultures. Semen collection efficiency, ejaculate traits and post-thaw semen quality, as evaluated by its in vitro survival rates, DNA damage and ATP concentration, were determined. Considering that the freezability of avian spermatozoa appears to be very different between species [15], even when showing very similar morphological shapes and ultrastructures [16,17], this information is fundamental in order to start establishing a semen cryobank of Griffon vultures. We took advantage of the presence of a local wildlife rescue centre where Griffon vultures are temporary recovered for sanitary problems and where a few wild-caught individuals originating from the natural population are permanently held in captivity because of unrecoverable traumas.

## Methods

### Chemicals

All reagents and media were from Sigma Co. (St. Louis, MO, USA) unless otherwise specified.

### Animals

This study was conducted from 2006 to 2008 during three consecutive reproductive seasons (December – March). The four individuals kept in captivity were sexed by the analysis of a sex related length polymorphism of the Chromosome helicase-DNA binding (CHD) gene present in most bird species [18], and were all classified as males. No female vulture was present in the wildlife rescue centre during this study. Age was estimated by plumage characteristics and by the data collected in their medical schedules. At the beginning of this study, males aged 25–30 (A), 11–13 (B), 8–10 (C), and 3 (D) years and their weight ranged form 6.7 to 8.1 kilos.

### Semen collection and evaluation

Semen collection attempts were made twice a week during the reproductive season (December – March) using the manual massage method [13]. The vulture was manually restrained during the semen collection procedure. A team of four people was required; two people held the wing and the head, the third person massaged simultaneously the bird's back and the mid abdomen to the vent. The massage was done for 2–3 minutes. As the vulture responded by everting its cloaca, the ejaculate was collected by the fourth person using a graduated pipette and its total volume was recorded. Urine or faecal contaminated ejaculates were discarded. Fresh semen was evaluated for sperm concentration using a haemocytometer and for viability by eosin-nigrosin stain [19]. The morphology of Griffon vulture spermatozoa was analyzed with an Olympus microscope equipped with a CCD camera and Olympus CellF software (2006, Olympus Soft Imaging Solutions GmbH). A total of 100 normal spermatozoa very randomly selected and analyzed for principal morphometric parameters (total, head, midpiece and tail length, maximum width).

### Semen cryopreservation

Only ejaculates showing more than 50% viable spermatozoa were cryopreserved. Semen samples were diluted 1:1 in TALP medium [13] supplemented with 20% egg yolk and cooled to 4°C, then an equal volume of TALP supplemented with 6% glycerol was added. After 20 minutes of equilibration at 4°C, semen was frozen in pellet form (0.05 ml) on dry ice and then plunged into liquid nitrogen. Thawing was carried out by plunging a sterilized glass falcon containing the pellet in a 39°C water bath for 20 s. After thawing, semen was washed in medium TALP by centrifugation at 800 rpm in a Eppendorf Microfuge (Eppendorf, Hamburg, Germany) for 3 minutes, and the pellet was re-suspended in the same medium.

### Post-thawing semen evaluation

In order to assess Griffon vulture semen cryotolerance and eventual differences in post-thawing semen quality among the four individuals, in vitro viability, DNA integrity and ATP concentration in frozen/thawed spermatozoa were evaluated.

#### In vitro viability assessment

In vitro viability was assessed during 4 hours in vitro culture in medium TALP at 38°C under 5% CO_2 _and maximum humidified atmosphere. Viability was assessed as previously described at 0, 1, 2, 3 and 4 hours of in vitro culture.

#### DNA integrity assessment

DNA damage of frozen/thawed Griffon spermatozoa was assessed by single-cell gel electrophoresis (comet assay). Analysis of the shape and length of "comet" tail, just like the DNA content in the tail, gives an assessment of DNA damage. The neutral comet assay was performed according to the method described by Sakkas et al. [20], with slight modifications. Briefly, sperm suspension (30 μl) was diluted in low-melting-point agarose (80 μl; 1% w/v). A 100-μl mixture of sperm-agarose was immediately pipetted onto 1% w/v normal-melting-point agarose-coated slides. Slides were immersed in ice-cold lysing solution (2.5 M NaCl, 100 mM EDTA, 10 mM Tris, 1% Triton X, and 10 mM dithiothreitol [DTT]; pH = 10) for 1 h at 4°C. Slides were then immersed in lysing solution supplemented with proteinase K (10 μg/ml). Incubation was performed during 1 h at 37°C. After this step, slides were rinsed in PBS and then placed in a horizontal electrophoresis tank filled with freshly prepared electrophoresis neutral buffer (Tris-acetate-EDTA [TAE], pH 7.3). Electrophoresis was performed at 10 V and 6 mA for 20 min. Following electrophoresis, the slides were neutralized with Tris-HCl buffer (pH 7.5) for 5 min and then fixed in methanol. Slides were stained with propidium iodide (PI), mounted with a coverslip and analyzed under an epifluorescence microscope. Digital comet images were captured with an Olympus microscope equipped with a CCD camera and Olympus CellF software. Fifty comets were measured per replicate sample (i.e., slide circle) using CASP software (Comet Assay Software Project 1.2.2). Scored parameters included tail DNA %, tail length (μm), Olive tail moment, and comet lenght. The Olive tail moment is a global comet parameter expressed as [(tail mean × head mean) × (% tail DNA/100)] and used to quantify DNA damage [21]. The comet length is defined as the distance in micrometers between the right and left edges of the comet.

#### ATP concentration determination

Each sample analyzed for ATP concentration was also assessed for spermatozoa viability, as previously described, in order to correlate these two variables.

Ten μl of both fresh and frozen/thawed spermatozoa (approximately 2 × 10^6 ^cells/ml, 20.000 total cells) were washed twice with 0,01 mL of cold physiological solution. For the extraction of nucleotides, 0.01 mL of ice-cold 0.6 M perchloric acid were added to each eppendorf containing spermatozoa and kept for 15 min; after the suspension was centrifuged in an Eppendorf Microfuge (3 minutes at 10000 rpm) and the supernatant was neutralized with 1.5 μL of 3.5 M K_2_CO_3 _[22]. After one successive centrifugation in a Eppendorf Microfuge (3 minutes at 10000 rpm), the supernatant was analyzed with capillary electrophoresis.

Sample derivatization was performed as described by Cornelius et al. [23] with some modifications. Briefly, 10 μL of sample or standard were mixed with 40 μL of 1.8 mol/L 1-ethyl-3-(3'-N,N'-dimethyl-aminopropyl)-carbodiimide (dissolved in 50 mM HEPES buffer, pH 6.5) and 5 μL of 27 mmol/L Bodipy FL EDA (dissolved in 50 mM HEPES buffer, pH 6.5) and incubated for 25 h at 37°C in the dark. Before analysis on capillary electrophoresis derivatized samples were diluted 40 fold in water. A P/ACE 5510 CE system equipped with Laser Induced Fluorescence (Beckman instruments, CA, USA) was used. The dimension of the uncoated fused-silica capillary was 75 μm ID and 57 cm length (50 cm to the detection window). Analysis was performed applying 21 nl of sample under nitrogen pressure (0.5 psi) for 3 s using a 10 mmol/L sodium phosphate buffer, pH 11.4. The separating conditions (22 kV at normal polarity) were reached in 20 s and held at a constant voltage for 8 min. All separations were carried out at 40°C.

### Statistical analysis

Statistical analysis were performed using the statistical software program Statgraphic Centurion XV (version 15.2.06 for Windows; StatPoint, Inc.) and a probability of P ≤ 0.05 was considered to be the minimum level of significance. One-way ANOVA was used to compare differences in ejaculate volume, semen concentration and viability, and ATP concentration among vultures. The Chi square test was used to compare semen collection efficiency (ejaculates obtained over total attempts) among males and sperm viability before and after cryopreservation. Differences in viability of frozen/thawed spermatozoa during 4 hours in vitro culture were determined by comparing viability regression lines using the "comparison of regression lines" function in the Statgraphic Centurion XV software. This function compares intercept (time: 0 to 4 hrs) and slope (viability rates) values of different lines showing how semen viability decrease during in vitro culture. Differences in comet assay scored parameters among males were compared by the analysis of variance for non parametric values (Kruskal-Wallis test), since data were not normally distributed (Levene's test: p < 0.001). ATP concentration and semen viability were correlated by the Multiple-Variable Analysis (Pearson test).

## Results

### Semen collection and evaluation

**Figure 1:**
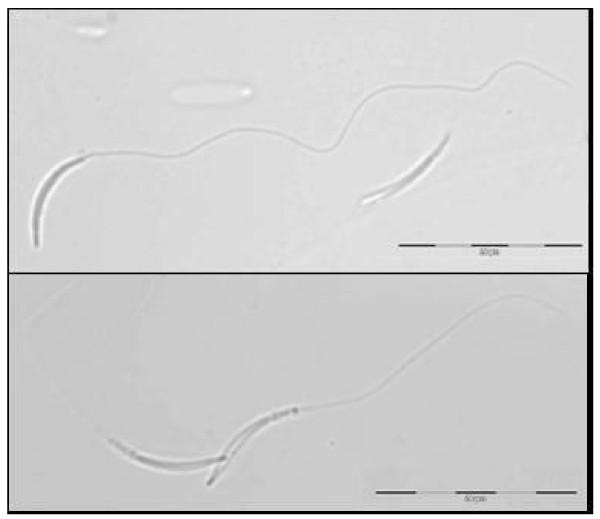
**Griffon vulture (*Gyps fulvus*) spermatozoa normal morphology**.

Griffon vulture spermatozoa are vermiform cells (figure 1) with a maximum width of 1 ± 0.2 μm and a length of 66 ± 8.7 μm (mean ± SD). The head is cylindrical and it is 9.2 ± 0.4 μm long; the midpiece is 2.6 ± 0.4 μm long and it is followed by a long flagellum, which accounts for approximately 86% of the cell's length (57 ± 6.7 μm).

Semen collection attempts were made during three consecutive reproductive seasons (December – March) using the manual massage method; ejaculation was successfully induced during a narrow window of time comprised between late January and late February. Semen collection efficiency, as calculated by the percentage of successful ejaculate induction over total attempts, was rather low (27.9%) and it did not vary among individuals (p > 0.05). In particular, out of 58 total attempts, 23 (39.6%) and 12 (20.7%) ejaculates were obtained from B and A, respectively. Since C was enrolled in this study at the end of the second reproductive season, it produced fewer ejaculates; out of 27 total attempts, 6 ejaculates (22.2%) were obtained. In order to assess if in the young male (D) spermatogenesis was at least commenced, urine/foecal samples collected during the abdominal massage were searched for spermatozoa; during the first two years no sperm cells were recorded, while in the third reproductive season 4 ejaculates were obtained out of 18 total attempts (22.2%). The Griffon Vulture is a long-lived species showing late sexual maturity (5–6 years old) and low fecundity [24]. Hence, the young male may have reached puberty in the third year of our research, i.e. at 6 years of age.

The mean volume of the ejaculates was rather small (12.5 ± 9.1 μl; table 1) and it did vary among the four vultures. Considering that spermatozoa mean concentration was 28.4 ± 30.9 × 10^6 ^cells/ml, total sperm output produced by the Griffon vultures in a single ejaculate was 355.000 ± 281.190 spermatozoa. No difference was found in spermatozoa concentration and viability (61.3 ± 13.9%) among the four vultures but these parameters showed a wide range of variability (table 1).

**Table 1:** Ejaculate volume, semen concentration and viability in four wild-caught Griffon vultures.

	Volume (μl)		Concentration (×10^6^/ml)		Viability (%)	
	Mean ± SD	Range	Mean ± SD	Range	Mean ± SD	Range
A	12.5 ± 6.3	6–20	32.9 ± 34	2–98.2	56.3 ± 12.4	42.9–75.9
B	13.4 ± 10.8	1–38	25.5 ± 21.6	3.8–76.7	63.8 ± 14.7	33.8–85.3
C	10.8 ± 7.3	3–20	38.6 ± 56.9	5–136.9	57.1 ± 14.7	41.1–70.2
D	11 ± 8.5	3–20	6.4 ± 5.7	2.4–10.5	61.2 ± 13.2	51.8–70.6
TOTAL	12.5 ± 9.1	1–38	28.4 ± 30.9	2–137.5	61.3 ± 13.9	33.8–85.2

### Post-thawing semen evaluation

Only ejaculates not being contaminated by faeces and/or urine and having more than 50% viable spermatozoa were cryopreserved. A high number of samples did not satisfy these criteria and had to be discarded; they accounted for the 66.7, 69.6, 50 and 25% of the total ejaculate obtained in A, B, C and D, respectively. Since it is considered imperative to preserve the unique genetic feature of each individual, we decided not to pool the samples before cryopreservation. Thus 4 ejaculates were cryopreserved from A (mean viability before freezing: 65.1 ± 3.4%), 7 from B (62.6 ± 2.9%), 3 from C (65 ± 7%) and 3 from D (61.5 ± 13.4%).

#### In vitro viability assessment

**Figure 2:**
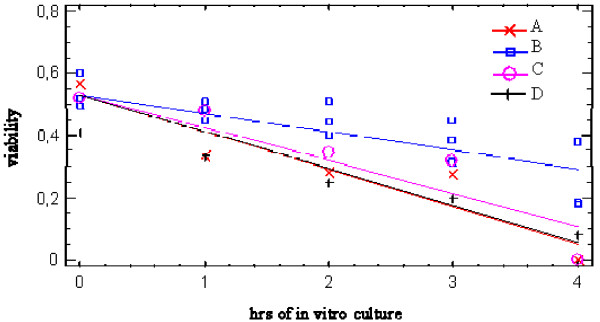
**Plot of fitted model showing viability decreasing rates over 4 hrs in vitro culture in frozen/thawed spermatozoa obtained from 4 Griffon vultures**. Comparison of regression lines: B ≠ A, C, D p < 0.001.

Spermatozoa viability after thawing did not differ between the four individuals (52.6 ± 5.8 in A, 53.4 ± 4.6 in B, 50.4 ± 3.2 in C, 42.5 ± 2.7 in D), but it decreased significantly compared to fresh semen (p < 0.05). As shown in figure 2, the analysis of the regression lines revealed that in the four vultures spermatozoa viability decreased following different rates (p < 0.001) over 4 hrs in vitro culture. In particular, spermatozoa collected from B maintained over time a higher viability in vitro when compared to A, C and D. On the other hand, no difference was found among A, C and D viability rates during in vitro culture.

#### DNA integrity assessment

**Figure 3:**
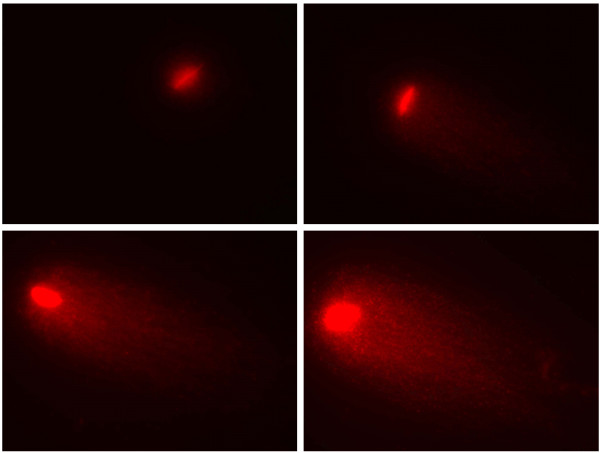
**Comet images of frozen/thawed Griffon vulture spermatozoa showing increasing levels of DNA damage**.

DNA fragmentation after freezing and thawing did non differ in the 4 vultures, as illustrated in table 2. The protocol described produced clear comet pictures (figure 3). Mean scored parameter did not differ significantly from values reported in literature for turkey spermatozoa after 2 hrs under refrigeration (4–7°C) [25].

**Table 2:** Differences in comet parameters in frozen/thawed spermatozoa collected from 4 Griffon vultures.

	Tail % DNA	Olive tail moment	Comet length (μm)
A	7 ± 8.5	19.9 ± 22.9	141.4 ± 46.9
B	16.3 ± 20.4	21.2 ± 26.9	166.9 ± 116.6
C	10.5 ± 12.8	20.9 ± 21.7	156.2 ± 83.4
D	9.7 ± 9.6	22.8 ± 23.7	155.8 ± 54.5
TOTAL	13 ± 17.1	21.3 ± 25.4	159.3 ± 94.8

Data are expressed as mean ± SD.

#### ATP concentration determination

The method used could determine ATP concentration in as little as 20.000 total sperm cells, thus allowing the performance of this assay in very small semen samples. Besides, there was a positive correlation between ATP concentration and semen viability (p < 0.05). Considering ejaculate small volume, this finding is of particular interested since it will allow to predict viability from ATP concentration, which is an objective and reliable parameter, and thus limit the number of assays needed to evaluate semen quality both before and after cryopreservation.

**Figure 4:**
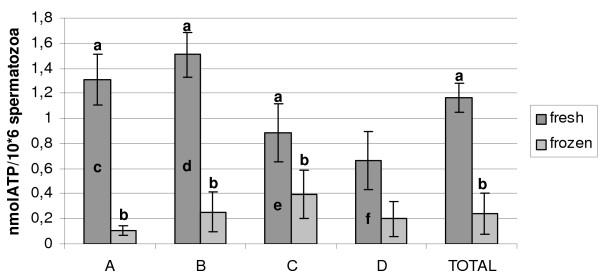
**ATP concentration (nmolATP/10^6 ^spermatozoa) in fresh and frozen/thawed spermatozoa collected from 4 Griffonvultures**. In the same group: Anova: a ≠ b p < 0.001; in different groups: Anova d ≠ e, f; c ≠ f p < 0.001.

Comparing ATP values in fresh semen among the four vultures, B showed higher nucleotide concentration than both C and D, while it did not differ form A, whose values were higher compared with D. Pooling the data obtained in the four vultures, ATP concentration in frozen/thawed semen was significantly lower than in fresh semen (p < 0.001); this result was confirmed even when analyzing nucleotide concentration in each individuals (p < 0.001), except for D. On the other hand, no difference was recorded in ATP concentration in frozen/thawed spermatozoa among the four individuals (figure 4).

## Discussion

This study reports original data on the basic reproductive parameters of the Griffon vulture, in terms of semen characteristics and ability to survive freezing and thawing procedures. Furthermore, a method to determine intracellular ATP concentration in as little as 20.000 total spermatozoa was used, allowing to perform this assay even in very small semen samples and thus to provide useful information about the energetic status of the sperm cells. Although the results reported here are based on a limited number of individuals, a common problem when dealing with endangered species, our data are important for the future development of genetic resource banks for this and related species. Besides, as part of this endeavour, it is essential to take the opportunity to learn about the basic reproductive biology of the species to be preserved.

The present study demonstrates that the abdominal massage method for semen collection can be used to induce ejaculation in the Griffon vulture, but the efficiency with which the vultures ejaculated (27.9%) was lower compared to the rates obtained in other birds, such as the Indian white-backed vulture [84.6%; [13]], the American kestrel [74%; [26]], the sandhill crane [86%; [27]], and the blue rock pigeon [90%; [28]], using the same method. Besides, we obtained ejaculates of rather small volumes (12.5 ± 9.1 μl) and with a high variability in sperm concentration (28.4 ± 30.9 spermatozoa × 10^6^/ml). In the Indian white-backed vulture (*Gyps bengalensis*), which is the closest specie we could refer to, ejaculate mean volume was 370 ± 360 μl, while average sperm concentration was 58.4 ± 33.2 spermatozoa × 10^6^/ml [13]. Total sperm output was therefore considerably lower in Griffon vultures used in the current study.

Since no female vulture was present in the wildlife rescue centre during this study, the use of visual contact with females in the case of Umapathy's study may explain the difference [29,30]. Researches on the role of behavioural stimuli in regulating the generation of mature gametes in birds are lacking. Cecil and Bakst [31] failed to find a positive influence of hen visual contact on ejaculate volume, concentration, and total spermatozoa output in male breeder turkeys. On the other hand, a more recent study reported that the confrontation with a female conspecific for 60 min positively affected plasma testosterone concentrations in male European starlings (*Sturnus vulgaris*) [30]. This finding agrees with the results of earlier studies [32,33]. It is generally recognized that testosterone plasma concentrations are positively related to sperm production since the hormone is needed for normal spermatogenesis and it is closely associated with testes size [for review see: [34]]. Differences in the behavioural response to human handling may also account for the low semen collection efficiency reported in this study for wild-caught Griffon vultures. Several methods have been used to obtain semen from birds: (1) killing and dissection of birds to remove sperm from the reproductive tract or testes [35]; (2) abdominal massage, which was developed by poultry biologist to obtain semen for artificial insemination [36,37]; (3) the application of a "sperm collector" to the cloaca [38]; (4) cooperative semen collection, in which imprinted birds voluntarily copulate on special devices in response to behavioural stimuli [39]; (5) use of a female dummy [40].

All of these techniques have limitations. The principal side effects of the abdominal massage method is that it is a stressful procedure, since it involves catching and handling of the birds, and that it stimulates urination, with consequent contamination of the ejaculates [10,39]. We had to discard the 62.2% of total ejaculates because of urine/faecal contamination or low viability, and this problem has already been described in other birds of prey [13,15].

Nevertheless we choose this method because it was the less invasive for not imprinted birds and it had been already tested on vultures [13]. Better results in terms of ejaculate induction in response to the stimulation and sperm output may be eventually obtained with birds raised in captivity and kept in visual contact with a female conspecific.

Considering that the Griffon vulture population in Sardinia dropped from 800 – 1200 to 100–140 individuals very rapidly and that there are no reports about the immigration of individuals from other colonies [14], we can also speculate that inbreeding depression consequent to isolation and small population size may account for the low semen output obtained in the current study. It is known that inbreeding reduces male reproductive function and semen quality [41,42]. In the endangered gazelle, *Gazella cuvieri*, the individual's coefficient of inbreeding correlated negatively with many parameters of semen quality, including ejaculate volume [43]. Unfortunately, information on genetic variability in the Sardinian Griffon vulture is lacking.

In order to assess Griffon vulture semen cryotolerance and eventual differences in post-thawing semen quality among the four individuals, in vitro viability, DNA integrity and ATP concentration in frozen/thawed spermatozoa were evaluated. Our data showed that spermatozoa viability after thawing did not differ between the four individuals, but it decreased significantly compared to fresh semen (p < 0.05).

Avian semen is particularly susceptible to freezing damage [6,44]. The high incidence of morphological disruption and ultrastructural abnormalities after cryopreservation have been related to its filiform shape and long tail which makes avian semen more susceptible to injury from mechanical manipulation [45,46]. Furthermore, poultry spermatozoa are more susceptible to osmotic stress that bovine spermatozoa [47] and their function is adversely impacted by excessive dilution [48]. Studies in chickens and turkeys have shown changes in many functions of spermatozoa, including viability, motility and ATP concentration after freezing [reviewed by refs. [44,49]].

In our study we reported that Griffon vulture spermatozoa can tolerate freezing and thawing procedure and are able to survive during 4 hrs in vitro culture. Noteworthy, after freezing/thawing spermatozoa collected from B maintained a higher viability during in vitro culture compared to spermatozoa from the other vultures.

Within-individual variation in the susceptibility of sperm cells to cryoinjury has been described in many different species [for review see: [50]]. Two biophysical traits of bird semen, i.e. resistance of spermatozoa to osmotic stress and membrane fluidity, have been reported to be possibly related to the differences between species and individual in the ability of spermatozoa to withstand the freezing-thawing process [51].

DNA fragmentation after freezing and thawing did non differ in the 4 vultures as evaluated by the comet assay method. The lack of repair mechanisms in sperm makes DNA damage irreversible. Griffon vultures frozen/thawed spermatozoa do not appear to be particularly susceptible to DNA fragmentation during cryopreservation since the mean scored parameters are similar to those recorded in turkey spermatozoa kept 2 hrs at 4–7°C [25]. Since cryopreservation may affect sperm function at many levels, in order to better assess Griffon vulture spermatozoa cryotolerance, adenosine triphosphate (ATP) intracellular concentrations were determined before and after cryopreservation. Individual differences in nucleotide levels in fresh semen were observed. This result is consistent with data reported for turkey spermatozoa, where most males segregate into characteristically high, average or low ATP categories [52]. Similarly a wide range of variability in ATP concentrations was described for fresh individual rooster semen [44,52]. Energy metabolism is a key factor supporting sperm function. Sustaining sperm motility and active protein modifications such as phosphorylation could be the reason why sperm require exceptionally more ATP than other cells [53]. The existence of different sperm mobility phenotypes in roosters has been already pointed out [54] and subsequently it has been related to differences in mitochondrial function [55]. In this study, an higher ATP concentration in fresh semen was followed by a longer survival in vitro after cryopreservation. Furthermore, its values correlated positively with sperm viability both before and after cryopreservation. It is generally accepted that assessment of the quality of fresh semen helps to predict the suitability of spermatozoa to withstand freezing and thawing. It would be interesting to investigate whether ATP intracellular concentration could be used as a tool to select the most suitable donor for semen cryopreservation in this and related species.

The extent of ATP loss in Griffon vulture spermatozoa was severe after cryopreservation. This result agrees with data reported for rooster [44] and crane spermatozoa (*Grus leucogeranus*) [56]. Given the apparent sensitivity of avian sperm mitochondria to the freeze/thaw process, it is not surprising that ATP production would be compromised after cryopreservation [44]. As a cause for low fertility of cryopreserved semen after artificial insemination, mitochondrial damage caused by freezing and thawing has been suggested [57,58]. Studies with isolated mitochondria proved that freezing and thawing largely impairs their bioenergetic functions [59]. These reports suggest that mitochondrial damage during cryopreservation is a major cause of low post thaw semen quality, but it is not adequately diagnosed with routine laboratory methods.

In conclusion, this study provides original information on Griffon vulture semen characteristic and cryotolerance, and points out the many difficulties that still prevent the widespread use of semen cryopreservation in the conservation of genetic resources in wild birds. Other techniques, such as the long-term preservation of primordial germ cells [60], the cryopreservation and transplantation of testicular tissue [61], and interspecies germ cell transplantation [62], are being studied but are not yet considered to be fully appropriate to the ex situ management of genetic resources. Semen cryopreservation may be considered as a useful tool in the management of captive populations of Griffon vultures, but further studies aimed to optimize semen collection efficiency in wild-caught birds and to test cryopreserved spermatozoa fertilizing potential are needed. Furthermore, accurate genetic analysis are needed to assess the genetic variability of the Griffon vulture Sardinian population.

## Competing interests

The authors declare that they have no competing interests.

## Authors' contributions

MM performed the semen collection and analysis, and helped drafting the manuscript. FB participated in the design of the study, carried out experimental analysis, performed the statistical analysis and drafted the manuscript. GGL participated in the design of the study and performed the statistical analysis. VS performed the DNA analysis for the individuation of the sex of the animals and participated in semen analysis. VP helped in performing the DNA and ATP analyses. ML, SS and AR helped in sample collection and in drafting of the manuscript and participated in the design of the study. AZ and CC performed the ATP analysis. MM conceptualised the idea, helped in drafting the manuscript and revising it critically for important intellectual content. SN conceived of the study, participated in its design and coordination and helped to draft the manuscript. All co-authors provided inputs during final manuscript preparation. All authors read and approved the final manuscript.

## References

[B1] Meuwissen TH, Sonesson AK (1998). Maximizing the response of selection with a predefined rate of inbreeding: overlapping generations. J Anim Sci.

[B2] Sonesson AK, Goddard ME, Meuwissen TH (2002). The use of frozen semen to minimize inbreeding in small populations. Genet Res.

[B3] Naitana S, Ledda S, Leoni G, Bogliolo L, Loi P, Cappai P (1998). Membrane integrity and fertilizing potential of cryopreserved spermatozoa in European mouflon. Anim Reprod Sci.

[B4] Berlinguer F, Leoni GG, Bogliolo L, Bebbere D, Succu S, Rosati I, Ledda S, Naitana S (2005). In vivo and in vitro fertilizing capacity of cryopreserved European mouflon (Ovis gmelini musimon) spermatozoa used to restore genetically rare and isolated populations. Theriogenology.

[B5] Berlinguer F, González R, Succu S, Del Olmo A, Garde JJ, Espes G, Gomendi M, Ledda S, Roldan ER (2008). In vitro oocyte maturation, fertilization and culture after ovum pick-up in an endangered gazelle (Gazella dama mhorr). Theriogenology.

[B6] Blesbois E, Grasseau I, Seigneurin F, Mignon-Grasteau S, Saint Jalme M, Mialon-Richard MM (2008). Predictors of success of semen cryopreservation in chickens. Theriogenology.

[B7] Saint Jalme M, Lecoq R, Seigneurin F, Blesbois E, Plouzeau E (2003). Cryopreservation of semen from endangered pheasants: the first step towards a cryobank for endangered avian species. Theriogenology.

[B8] Blesbois E, Seigneurin F, Grasseau I, Limouzin C, Besnard J, Gourichon D, Coquerelle G, Rault P, Tixier-Boichard M (2007). Semen cryopreservation for ex situ management of genetic diversity in chicken: creation of the French avian cryobank. Poult Sci.

[B9] Brock MK, Bird DM (1991). Prefreeze and post-thaw effects of glycerol and dimethylacetamide on motility and fertilizing ability of American kestrel (Falco sparverius) spermatozoa. J Zoo Wildl Med.

[B10] Gee GF, Bakst MR, Sexton TJ (1985). Cryogenic preservation of semen from the Greater Sandhill Crane. J Wildl Manage.

[B11] Gee GF, Sexton TJ (1990). Cryogenic preservation of semen from the Aleutian Canada Goose (Branta canadensis leucopareia). Zoo Biol.

[B12] Samour H, Markham JA, Moore HDM (1988). Semen cryopreservation and artificial insemination in budgerigars (Melopsittacus undulatus). J Zool.

[B13] Umapathy G, Sontakke S, Reddy A, Ahmed S, Shivaji S (2005). Semen characteristics of the captive Indian white-backed vulture (Gyps bengalensis). Biol Reprod.

[B14] Schenk H, Aresu M, Naitana S (2005). Gyps fulvus Regional Action Plan. Rivista della Federazione Italiana Parchi e delle Riserve Naturali.

[B15] Blanco JM, Gee G, Wildt DE, Donoghue AM (2000). Species variation in osmotic, cryoprotectant, and cooling rate tolerance in poultry, eagle, and peregrine falcon spermatozoa. Biol Reprod.

[B16] Massip A, Leibo S, Blesbois E, Benson E, Fuller B, Lane N (2004). Cryobiology and the breeding of domestic animals. Life in the frozen state.

[B17] Blesbois E, Labbe' C, Planchenault D (2003). Main improvements in semen and embryo cryopreservation for fish and fowl. Cryopreservation of Animal Genetic Resources in Europe.

[B18] Griffiths R, Double MC, Orr K, Dawson RGJ (1998). A DNA test to sex most birds. Mol Ecol.

[B19] Pintado B, de la Fuente J, Roldan ER (3258). Permeability of boar and bull spermatozoa to the nucleic acid stains propidium iodide or Hoechst 3 or to eosin: accuracy in the assessment of cell viability. J Reprod Fertil.

[B20] Sakkas D, Moffatt O, Manicardi GC, Mariethoz E, Tarozzi N, Bizzarro D (2002). Nature of DNA damage in ejaculated human spermatozoa and the possibile involvement of apoptosis. Biol Reprod.

[B21] Olive PL, Durand RE, Banath JP, Johnston PJ (2001). Analysis of DNA damage in individual cells. Methods Cell Biol.

[B22] Balestri F, Giannecchini M, Sgarrella F, Carta MC, Tozzi MG, Camici M (2007). Purine and pyrimidine nucleosides preserve human astrocytoma cell adenylate energy charge under ischemic conditions. Neurochem Int.

[B23] Cornelius M, Wörth CG, Kliem HC, Wiessler M, Schmeiser HH (2005). Detection and separation of nucleoside-5'-monophosphates of DNA by conjugation with the fluorescent dye BODIPY and capillary electrophoresis with laser-induced fluorescence detection. Electrophoresis.

[B24] Sarrazin F, Cagnolini C, Pinna JL, Danchin E (1996). Breeding biology during establishment of a reintroduced Griffon vulture Gyps fulvus population. Ibis.

[B25] Kotłowska M, Dietrich G, Wojtczak M, Karol H, Ciereszko A (2007). Effects of liquid storage on amidase activity, DNA fragmentation and motility of turkey spermatozoa. Theriogenology.

[B26] Bird DM, Lague PC, Buckland RB (1976). Artificial insemination vs natural mating in captive American kestrels. Can J Zool.

[B27] Gee GF, Temple SA (1978). Artificial insemination for breeding nondomestic birds. Symp Zool Soc Lond.

[B28] Sontakke SD, Umapathy G, Sivaram V, Kholkute SD, Shivaji S (2004). Semen characteristics, cryopreservation, and successful artificial insemination in the Blue rock pigeon (Columba livia). Theriogenology.

[B29] Bozynski CC, Liley NR (2003). The effect of female presence on spermiation, and of male sexual activity on 'ready' sperm in the male guppy. Anim Behav.

[B30] Pinxten R, de Ridder E, Eens M (2003). Female presence affects male behavior and testosterone levels in the European starling (Sturnus vulgaris). Horm Behav.

[B31] Cecil HC, Bakst MR (1990). Effect of the presence of hens on the semen production of male breeder turkeys. Poult Sci.

[B32] Feder HH, Storey A, Goodwin D, Reboulleau C, Silver R (1977). Testosterone and 5α-dihydrotestosterone levels in peripheral plasma of male and female ring doves (Streptopelia risoria) during the reproductive cycle. Biol Reprod.

[B33] Dufty AM, Wingfield JC (1986). The influence of social cues on the reproductive endocrinology of male brown-headed cowbirds: field and laboratory studies. Horm Behav.

[B34] Kirby JD, Froman DP, Whittow GC (2000). Reproduction in male birds. Sturkie's Avian Physiology.

[B35] Henley C, Feduccia A, Costello DP (1978). Oscine spermatozoa: a light- and electron-microscopy study. Condor.

[B36] Burrows WH, Quinn JP (1937). The collection of spermatozoa from the domestic fowl and turkey. Poultry Sci.

[B37] Samour J, Smith CA, Moore HDM, Markham J (1986). Semen collection and spermatozoa characteristics in Budgerigars (Melopsittacus undulatus). Vet Rec.

[B38] Pizzari T, Birkhead TR (2000). Female feral fowl eject sperm of subdominant males. Nature.

[B39] Blanco JM, Gee GF, Wildt DE, Donoghue AM (2002). Producing progeny from endangered birds of prey: treatment of urine-contaminated semen and a novel intramagnal insemination approach. J Zoo Wildl Med.

[B40] Rybnik PK, Horbanczuk JO, Naranowicz H, Lukaszewicz E, Malecki IA (2007). Semen collection in the ostrich (Struthio camelus) using a dummy or a teaser female. Br Poult Sci.

[B41] van Eldik P, Waaij EH van der, Ducro B, Kooper AW, Stout TA, Colenbrander B (2006). Possible negative effects of inbreeding on semen quality in Shetland pony stallions. Theriogenology.

[B42] Gomendio M, Cassinello J, Roldan ER (2000). A comparative study of ejaculate traits in three endangered ungulates with different levels of inbreeding: fluctuating asymmetry as an indicator of reproductive and genetic stress. Proc Biol Sci.

[B43] Roldan ERS, Cassinello J, Abaigar T, Gomendio M (1998). Inbreeding, fluctuating asymmetry, and ejaculate quality in an endangered ungulate. Proc Biol Sci.

[B44] Long JA (2006). Avian Semen Cryopreservation: What Are the Biological Challenges?. Poult Sci.

[B45] Donoghue AM, Wishart GJ (2000). Storage of poultry semen. Anim Reprod Sci.

[B46] Agca Y, Critser JK (2002). Cryopreservation of spermatozoa in assisted reproduction. Semin Reprod Med.

[B47] Watson PF, Kunze E, Cramer P, Hammerstedt RH (1992). A comparison of critical osmolality and hydraulic conductivity and its activation energy in fowl and bull spermatozoa. J Androl.

[B48] Bakst MR, Crawford RD (1990). Preservation of avian cells. Poultry Breeding and Genetics.

[B49] Blesbois E (2007). Current status in avian semen cryopreservation. World's Poult Sci J.

[B50] Thurston LM, Watson PF, Holt WV (2002). Semen cryopreservation: a genetic explanation for species and individual variation?. Cryo Letters.

[B51] Blesbois E, Grasseau I, Seigneurin F (2005). Membrane fluidity and the ability to survive cryopreservation in domestic bird spermatozoa. Reproduction.

[B52] Long JA, Guthrie HD (2006). Validation of a rapid, large-scale assay to quantify ATP concentration in spermatozoa. Theriogenology.

[B53] Miki K (2007). Energy metabolism and sperm function. Soc Reprod Fertil Suppl.

[B54] Froman DP, Feltmann AJ, Rhoads ML, Kirby JD (1999). Sperm mobility: a primary determinant of fertility in the domestic fowl (Gallus domesticus). Biol Reprod.

[B55] Froman DP, Kirby JD (2005). Sperm Mobility: Phenotype in Roosters (Gallus domesticus) Determined by Mitochondrial Function. Biol Reprod.

[B56] Maksudov GIU, Erokhin AS, Nesterenko ON, Panchenko VG (2002). ATP content in cryopreserved sperm of Siberian white cranes Grus leucogeranus (Aves: Gruiformes). Izv Akad Nauk Ser Biol.

[B57] Ruiz-Pesini E, Diez C, Lapena AC, Perez-Martos A, Montoya J, Alvarez E (1998). Correlation of sperm motility with mitochondrial enzymatic activities. Clin Chem.

[B58] Schober D, Aurich C, Nohl H, Gille L (2007). Influence of cryopreservation on mitochondrial functions in equine spermatozoa. Theriogenology.

[B59] Mori Y, Suzuki H, Nei T (1986). Freezing injury in the yeast respiratory system. Cryobiology.

[B60] Tajima A, Naito M, Yasuda Y, Kuwana T (1998). Production of germ-line chimeras by transfer of cryopreserved gonadal primordial germ cells (gPGCs) in chicken. J Exp Zool.

[B61] Song Y, Silversides FG (2007). Production of Offspring from Cryopreserved Chicken Testicular Tissue. Poult Sci.

[B62] Kang SJ, Choi JW, Kim SY, Park KJ, Kim TM, Lee YM, Kim H, Lim JM, Han JY (2008). Reproduction of wild birds via interspecies germ cell transplantation. Biol Reprod.

